# Bouveret’s syndrome as a rare complication of cholelithiasis: Disputes in current management and report of two cases

**DOI:** 10.1016/j.ijscr.2020.05.019

**Published:** 2020-05-25

**Authors:** Giuseppe Evola, Sebastiano Caramma, Giovambattista Caruso, Giovanni Dapri, Francesco Roberto Evola, Carlo Reina, Giuseppe Angelo Reina

**Affiliations:** a General and Emergency Surgery Department, Garibaldi Hospital, Catania, Italy; b General Surgery Department, San Salvatore Hospital, Paternò, Catania, Italy; c Department of Gastrointestinal Surgery, Saint-Pierre University Hospital, Brussels, Belgium; d Department of Orthopedic and Traumatology, Cannizzaro Hospital, Catania, Italy

**Keywords:** Bouveret’s syndrome, Cholelithiasis, Cholecysto-duodenal fistula, Gallstone ileus, Gastrotomy, Case report

## Abstract

**Introduction:**

Bouveret’s syndrome is a rare complication of cholelithiasis that determines an unusual type of gallstone ileus, secondary to an acquired fistula between the gallbladder and either the duodenum or stomach with impaction of a large gallbladder stone. Preoperative diagnosis is difficult because of its rarity and the absence of typical symptoms. Adequate treatment consists of endoscopic or surgical removal of obstructive stone.

**Presentation of cases:**

Two old females patients were admitted to the Emergency Department with a history of abdominal pain associated with bilious vomiting. Physical examination revealed abdominal distension with tympanic percussion of the upper quadrants, abdominal pain on deep palpation of all quadrants and in the first patient positive Murphy’s sign. Preoperative diagnosis of gallstone impacted in the duodenum was obtained by abdominal computed tomography (CT) scan in the first patient and by esophagogastroduodenoscopy in the second one. Both patients underwent surgery with extraction of the gallstone from the stomach. Postoperative course of two patients was uneventful and they were discharged home.

**Discussion:**

Bouveret’s syndrome usually presents with signs and symptoms of gastric outlet obstruction. Preoperative radiological investigations not always are useful for its diagnosis. Appropriate treatment, endoscopic or surgical, is debated and must be tailored to each patient considering medical condition, age and comorbidities.

**Conclusion:**

Bouveret’s syndrome is a very rare complication of cholelithiasis, difficult to diagnose and suspect, because of lack of pathognomonic symptoms. Nowaday there are no guidelines for the correct management of this pathology. Endoscopic or surgical removal of obstructive stone represents the correct treatment.

## Highlights

• Bouveret’s syndrome is a rare complication of cholelithiasis that determines an unusual type of gallstone ileus.
• This syndrome is associated with high morbility and mortality rates.
• Diagnosis of Bouveret’s syndrome is difficult because of non-specific symptoms and its rarity.
• Here are no guidelines for the diagnostic work up and the correct management of this pathology.
• The endoscopic or surgical management should be tailored to the patient’s clinical presentation and morbidities.

## 1 Introduction

Bouveret’s syndrome is a rare cause of gastric outlet obstruction caused by impaction of a large gallbladder stone in the duodenum or pylorus. It represents a rare complication of cholelithiasis (0.3–0.5% of cases) that determines an unusual type of gallstone ileus (1–3% of cases) [1]. This syndrome is characterized by the passage of a large gallstone through a cholecysto-gastric or cholecysto-duodenal fistula in the stomach or duodenal bulb (as in our cases) resulting in mechanical obstruction [2]. It can affect any age group (25–91 years) and is most common in elderly women [3] with medical comorbidities and a history of cholelithiasis. Diagnosis depends on the non specificity of its symptoms and its rarity. Treatment consists of endoscopic [4] or surgical removal of obstructive stone. Mortality rate has decreased in recent years [5] but remains high due to the frequent delayed and overlooked diagnosis and to the numerous comorbidities affecting patients [4]. Two cases of Bouveret’s syndrome are presented with review of the literature, in accordance with the Surgical Case Reports (SCARE) criteria [6].

## 2 Presentation of cases

### 2.1 Case 1

**Fig. 1:**
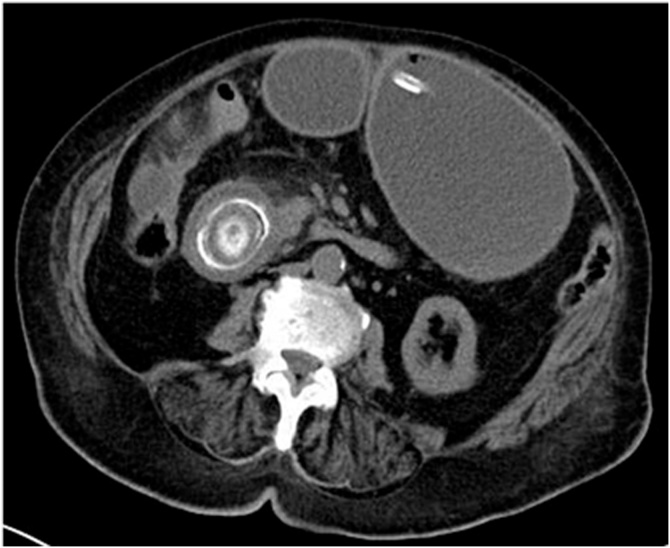
Abdominal CT scan showing a dilated stomach and a calcified stone impacted in the second portion of duodenum.

**Fig. 2:**
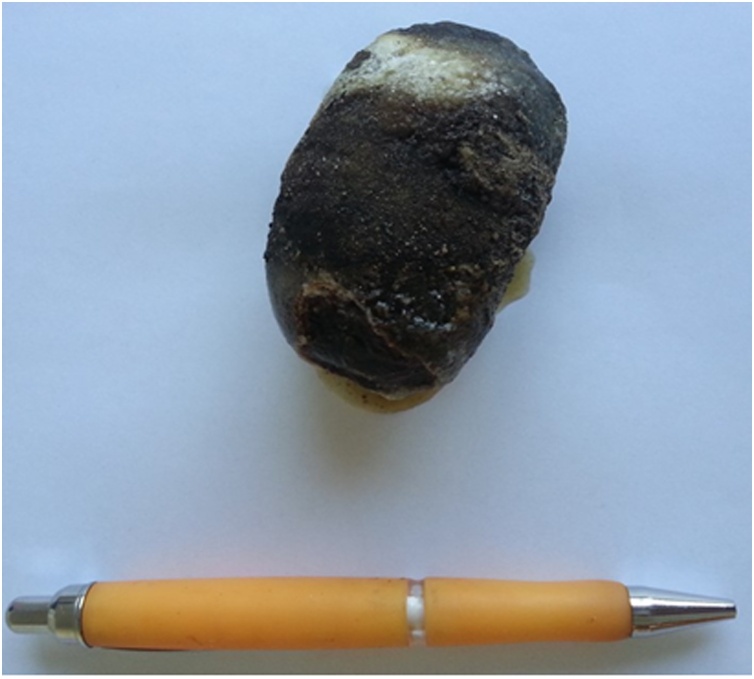
Gallstone impacted in the duodenum measuring 7 × 3.5 cm.

A 85-year-old Caucasian female was admitted to the Emergency Department with a ten-day history of abdominal pain and constipation and a one-day history of bilious vomiting. Her past medical history included arterial hypertension, type 2 diabetes mellitus and sideropenic anemia; vital signs were normal. Physical examination revealed abdominal distension with tympanic percussion of the upper quadrants and abdominal pain on deep palpation of all quadrants with positive Murphy’s sign. Laboratory examinations reported neutrophilic leukocytosis (WBC 18.800 × 10^3^/μL) and anemia (hemoglobin 8.4 g/dl). Abdominal computed tomography (CT) scan showed the presence of a dilated stomach and a large stone impacted in the duodenum (Fig. 1), caused by a cholecysto-duodenal fistula, without the presence of further gallstone in the gallbladder. A nasogastric tube was placed and drained two liters of gastric contents; the patient was treated with intravenous antibiotics. Cardiologic and anesthesiological examinations classified the patient as American Society of Anesthesiologist physical classification grade III (ASA III). At midline laparotomy the gallbladder appeared inflamed and fused to the duodenum. After kocherization of the duodenum a gastrotomy was performed by trans-gastric extraction of the large stone, moved manually into the stomach. The stone measured 7 × 3.5 cm (Fig. 2). The stomach was closed by firings of linear mechanical stapler. The postoperative course was uneventful, the patient was discharged on 7^th^ postoperative day. After eight months, the patient is asymptomatic and well.

### 2.2 Case 2

**Fig. 3:**
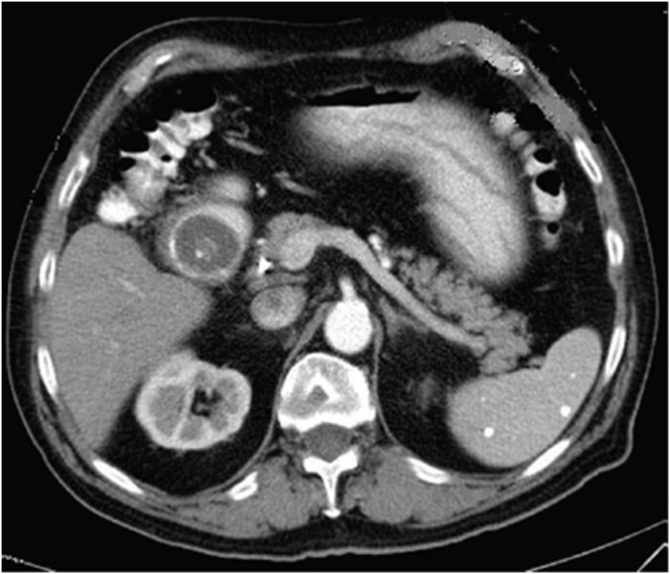
Abdominal CT scan showing a subtotal stenosis of the lumen of the second duodenal portion.

**Fig. 4:**
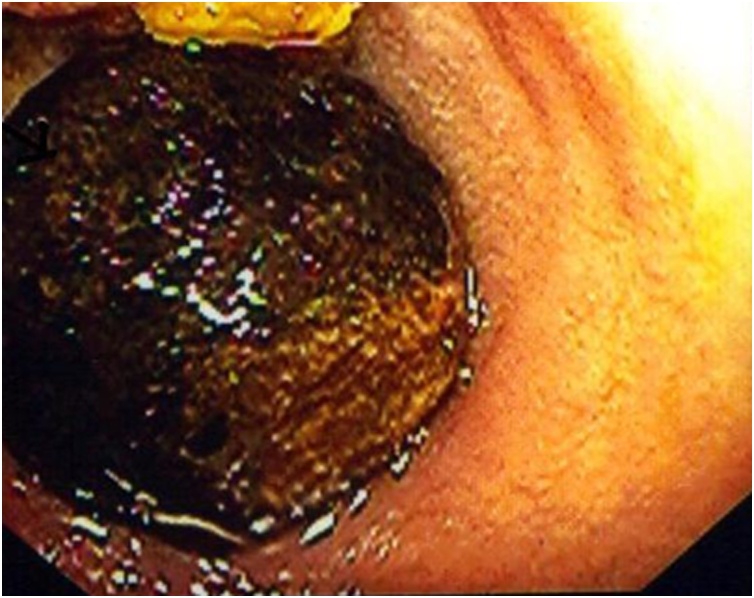
Upper endoscopy demonstrates a large, round, smooth mass occupying alomst the entire duodenal lumen.

**Fig. 5:**
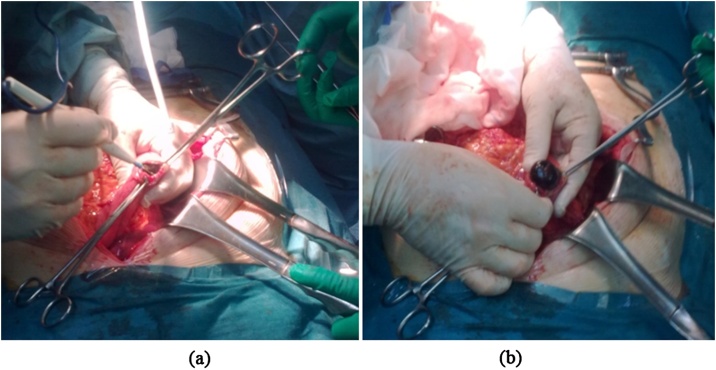
(a) Gastrotomy at the antrum. (b) Extraction of the gallstone from the stomach.

A 79-year-old Caucasian female was admitted to the Emergency Department with a two-day history of abdominal pain, nausea and bilious vomiting. Her past medical history included arterial hypertension, type 2 diabetes mellitus and hysteroannessiectomy; vital signs were normal. Physical examination revealed abdominal distension with tympanic percussion of the upper quadrants and abdominal pain on deep palpation of the epigastrium without obvious muscle guarding or rebound tenderness. Laboratory tests reported a normal white cell count, C-reactive protein level of 37.10 mg/L (reference range <7.5 mg/L), total bilirubin level of 2.0 mg/dL (reference range <1.2 mg/dL) and direct bilirubin level of 0.8 mg/dL (reference range <0.3 mg/dL). Abdominal CT scan showed the presence of a subtotal stenosis of the lumen of the second duodenal portion caused by a nodular lesion (Fig. 3) with concomitant gastric distension. Esophagogastroduodenoscopy revealed a large, round, smooth, non-friable and non-fleshy mass (gallstone) occupying almost the entire duodenal lumen (Fig. 4). Endoscopic retrieval of the gallstone failed. The patient was classified as ASA III and underwent laparotomy. Intraoperatively, the gallbladder was found to be adherent to the duodenum and a gallstone was found impacted in the second part of the duodenum and was manually moved into the stomach to be retrieved by a gastrotomy (Fig. 5). The size of extracted stone was 3.5 × 5 cm. Inspection and palpation of the duodenum revealed the presence of a cholecysto-duodenal fistula not identified preoperatively. The patient was discharged on 8^th^ postoperative day and after a follow-up of six months is asymptomatic.

## 3 Discussion

These clinical cases compare our experience with literature data. This syndrome is difficult to diagnose because of non-specific symptoms and its rarity, however a high clinical suspicion and the aid of imaging studies can make the diagnosis easier. Different radiological exams are useful for diagnosis like abdominal plain X-ray (AXR), abdominal ultrasonography (US), abdominal CT scan and magnetic resonance imaging (MRI). Findings suggestive of Bouveret’s syndrome on AXR include Rigler’s triad (air within the biliary tree, distendend stomach and ectopic radio-opaque gallstones) [7]. Rigler’s triad is pathognomonic but only 14–53% of cases present with the full criteria on AXR [8]; in our first case report Rigler’s triad was absent on AXR. US identifies the Rigler’s triad in 11% of cases and CT scan can identify up to 80% of cases [9]. MRI can demonstrate Rigler’s triad in almost 100% of cases [8]. Esophagogastroduodenoscopy may reveal gastro-duodenal obstruction in all cases, however it shows the obstructing stone in 69% of cases and fistulous stoma in 13% of examinations [10]. Although literature reports a preoperative diagnosis in 50% of cases [11], in both our cases diagnosis was obtained preoperatively: in the first case by abdominal CT scan and in the second case by esophagogastroduodenoscopy. Because of the low incidence, uniform management has not been clearly defined and a debate exists with regard the endoscopic or surgical treatment. Endoscopic retrieval of the stones has the benefits of minimal access approach, however its success depends on the presence of adequate endoscopic skills and if the stone is small [12]. In our clinical cases, according to the literature [13], both gallstones impacted in the duodenum were greater than 3.5 cm and endoscopic treatment in the second case failed. Complications of endoscopic treatment are stone impaction in the esophagus, distal gallstone ileus, gastrointestinal hemorrhage or perforation [14]. Despite endoscopic treatment, 91% of patients need surgery [7]. Surgery is the mainstay treatment in situations such as stone impaction in the fistula, stone compression of the duodenal wall and gastrointestinal bleeding [15]. Surgical options include gastrotomy (or duodenotomy), gastrotomy (or duodenotomy) with cholecystectomy and fistula closure (one-stage procedure) and gastrotomy (or duodenotomy) with cholecystectomy and repair of the bilio-digestive fistula after 4–6 weeks (two-stage procedure) [2]. A debate exists whether cholecystectomy with fistula repair should be carried out at the same time as the relief of gastric outlet obstruction, performed later or not at all [2]. Gastrotomy is the procedure of choice, as in our cases, if the stone is located inside the stomach or if it is impacted inside the duodenum and can be maneuvered into the gastric lumen, but some groups advocate concomitant cholecystectomy and definitive correction of the internal fistula to prevent biliary complications [14]. Cholecystectomy is indicated for retained gallstones in the gallbladder to prevent recurrence and biliary complications [16]. Only 10% of patients require delayed cholecystectomy with fistula repair for persistent biliary symptoms [11]. In our clinical cases no patient required further surgical treatment after stone extraction alone. A retrospective review about 3268 gallstone ileus cases reported an overall mortality rate of 6.67%: mortality rates were 4.94% for the enterolitotomy alone group and 7.25% for enterolithotomy plus cholecystectomy and fistula repair group [16]. A tailored surgical treatment is the key to successful management: stone extraction alone should be advised in elderly patients with associated comorbidities (ASA III-IV) and in case of recurrent biliary complications a subsequent cholecystectomy with fistula repair is indicated; one-stage procedure with fistula repair and cholecystectomy should be offered only to selected young patients in good general condition (ASA I-II) [17]. Our patients, classified as ASA III, accepted surgery and underwent stone extraction alone. In older and comorbid patients in acceptable condition with non acute inflammatory tissue conditions at surgery, one-stage procedure may be considered to avoid secondary complications [7]. Although experience in minimally invasive surgery of gallstone ileus is still developing, adequate management in low risk patients has allowed successful results [2]; however laparoscopy, reported as safe and effective [7], is used only in 10% of surgically managed gallstone ileus cases, with a high conversion rate to laparotomy [16]. The mortality rate of Bouveret’s syndrome in the past has reached 30%–50% of cases but in recent years has improved to about 12% [15], however it remains high because of the elderly age/multiple comorbidities of the patients and the difficult diagnosis. Both patients are in good health without complications related to restrictive surgery.

## 4 Conclusion

Bouveret’s syndrome is a rare complication of cholelithiasis that usually presents with signs and symptoms of gastric outlet obstruction. Because of the non specificity of its symptoms and its rarity, the diagnosis is difficult and often delayed or overlooked. The awareness of this entity and a high index of suspicion are required to make an early diagnosis and to initiate the appropriate endoscopic and/or surgical treatment. Nowaday there are no standardized recommendations for the diagnostic work up and guidelines for the correct management of this pathology associated with high morbility and mortality rates. The best approach is the one tailored to each patient considering medical condition, age, comorbidities, life expectancy and surgeon’s experience.

## Declaration of Competing Interest

All the authors certify that there is no conflict of interest regarding the material discussed in the manuscript.

## Funding

All the authors declare that this research didn’t receive any specific grant from funding agencies in the public, commercial, or not-for-profit sectors.

## Ethical approval

Ethical approval has been exempted by our institution because this is a case report and no new studies or new techniques were carried out.

## Consent

Written informed consent was obtained from the patients for publication of this case report and accompanying images.

## Author’s contribution

Giuseppe Evola: Drafting the manuscript and literature research.

Sebastiano Caramma: Drafting the manuscript and literature research.

Giovambattista Caruso: Operated on the patients, literature research.

Giovanni Dapri: Revising the manuscript.

Francesco Roberto Evola: Drafting the manuscript and literature research.

Carlo Reina: Drafting the manuscript and literature research.

Giuseppe Angelo Reina: Operated on the patients, revising the manuscript.

## Registration of research studies

This case report does not require registration as a research study.

## Guarantor

The guarantor for this case report is Giuseppe Evola.

## Provenance and peer review

Not commissioned, externally peer-reviewed.
